# Expression of transcription factors snail, slug, and twist in human bladder carcinoma

**DOI:** 10.1186/1756-9966-29-119

**Published:** 2010-09-01

**Authors:** Qinchao Yu, Kejun Zhang, Xinsheng Wang, Xiangping Liu, Zemi Zhang

**Affiliations:** 1 Department of Urology surgery, the Affiliated Hospital of medical college, QingDao University, QingDao, Shan Dong Province, 266403, China; 2 Department of General surgery, the Affiliated Hospital of medical college, QingDao University, QingDao, Shan Dong Province, 266403, China; 3 Department of molecular biology, the Affiliated Hospital of medical college, QingDao University, QingDao, Shan Dong Province, 266403, China

## Abstract

**Background:**

Slug, Snail, and Twist are transcription factors that regulate the expression of tumor suppressors such as E-cadherin. In this study, we aimed to examine the expression of these transcription factors in human bladder carcinoma.

**Methods:**

We first investigated expression of Slug, Snail, Twist and E-cadherin in five bladder Carcinoma cell lines by reverse transcription-polymerase chain reaction and western blotting. Furthermore, we investigated Slug, Snail, and Twist and E-cadherin expression by immunohistochemistry with bladder carcinoma (tumor, n = 120; background, n = 42).

**Results:**

Expression of Slug mRNA and protein was detected in all cell lines, Twist was clearly expressed in two out of five bladder carcinoma cell lines, Snail was not expressed, and E-cadherin was detected in 3 cell lines. 44.2% (53/120) of human bladder Carcinoma tissues and 38%(16/42) background tissue showed an expression of Twist; 62.5%(75/120) of human bladder Carcinoma tissues and 40%(17/42) background tissue showed an expression of Slug, 15.8% (19/120) of human bladder Carcinoma tissues and 76%(32/42) background tissue showed an expression of Snail, and 25.8% (31/120) cases were negative for E-cadherin expression in carcinoma tissues. Expression of Slug and Twist shows increased levels in tumors, whereas Snail seems reduced. Statistically significant correlations were found between Twist, Slug, and E-cadherin expression. Immunohistochemistry analysis showed that Twist was elevated with increasing tumor stage (*P *= 0.001), the grade (*P *< 0.001), the progression (*P *= 0.035). Slug was elevated and Snail was reduced with increasing nodal involvement (tumor-node-metastasis status) (*P *= 0.004, *P *= 0.01). E-cadherin was reduced in expression corresponding with tumor grade (*P *< 0.01). Positive Twist, Slug and E-cadherin expression clearly predicted poorer PFS (*P *= 0.042, *P *= 0.014, *P *= 0.001). In the multivariate analysis, only Snail and E-cadherin expression were independent prognostic factors for OS (P = 0.002, *P *< 0.001).

**Conclusions:**

These data demonstrate that Twist, Snail and Slug have inappropriate expression in bladder carcinoma and that this may play a part in the progression of human bladder carcinoma.

## Background

Bladder cancer is the second most common genitourinary tract cancer and the fourth or fifth most common cancer of men in western industrialized countries[1]. In China, bladder cancer is the most common malignancy in genitourinary tract and the fifth most common cancer in men. Generally, radical cystectomy is considered the standard treatment for patients with muscle-invasive tumors, and systemic chemotherapy is the only current modality that provides the potential for long-term survival in patients with metastatic disease, but the prognosis of patients with advanced bladder cancer is still extremely poor despite recent therapeutic advances[2]. It is well known that bladder tumors with the same stage and grade have a heterogeneous clinicaloutcome as they are probably molecularly different. The term of superficial or invasive bladder tumor is confusing as it implies that only one kind of superficial or invasive bladder cancer exists[3]. Understanding the molecular biology of bladder cancer and metastasis may provide insight for the development of novel tumor markers or new therapeutic strategies.

Epithelial-mesenchymal transition (EMT) has emerged as a critical process during cancer progression in which downregulation or loss of E-cadherin expression (epithelial marker) constitute a molecular hallmark [4,5]. The transcriptional factors Snail and Slug (zinc finger proteins) have been described to be direct repressors of E-cadherin [6-11]*in vitro *and *in vivo *through an interaction of their COOH-terminal region with a 5'-CACCTG-3' sequence in the E-cadherin promoter [12]. Both have been suggested to be involved in the acquisition of resistance to apoptosis, thereby promoting tumor survival. Recently, it has been postulated that Twist, another promoter repressor of CDH1 (E-cadherin gene), may be involved in tumor progression by silencing E-cadherin expression and EMT induction [13,14]. Twist is considered as a promoter of the EMT, which is a key event in the tumoral invasion step. Up-regulation of Twist is associated with malignant transformation of melanoma and T-cell lymphoma [13]. It is possibly involved in E-cadherin conversion during EMT [14]. Studies in other cancers have shown that overexpression of Snail and Slug leads to a reduction of E-cadherin expression. An overexpression of Twist resulted in an a further decrease of E-cadherin expression [15].

Because Snail, Twist and Slug are potential regulators of cell adhesion and migration, this study aimed to determine the levels of expression of Snail, Slug, and Twist in human bladdert cancer tissues and to elucidate whether these levels are clinically significant. Also, to clarify whether the three factors may be used as a novel parameter to predict prognosis in bladder carcinoma.

## Materials and methods

### Patients and paraffin-embedded tissue sample

The study included 120 patients with a primary bladder tumor and 42 background tissue(paracarcinoma tissue, more than 1.5-2 cm from cancer tissue). The tissues were obtained from patients who had undergone a transurethral resection or a partial/total cystectomy between 1999 and 2002 at the Urology Department, The affiliated hospital of Qingdao medical college, Qingdao university, China. None of the patients had received preoperative treatment. All patients were classified according to the 1997 UICC TNM classification for the stage and OMS 2004 for the grade (LMP: low malignant potential; LG: low grade; HG: high grade). Immunostaining was evaluated by 2 independent pathologists to validate the diagnosis. Each sample was used after written consent was obtained from the patients. The clinicopathological data of the tumors are shown in Table 1. Tumor recurrence/progression was defined based on clinical, radiological, or histological diagnoses. The study was approved by the affiliated hospital of Qingdao medical college Faculty of Medicine Human Investigation Committee.

**Table 1:** Clinical information of patient samples analyzed

Variable	n (%)
Tissue type	
Background	42
Tumor	120
Age - yr (mean)	
< 70	64 (53)
≥70	56 (47)
Gender - number of patients	
Male	87 (72)
Female	33 (28)
Grade - no. of patients	
LG	41 (34)
HG	79 (66)
Stage - number of patients (%)	
NMIBC:	
Ta	31 (26)
T1	45 (37)
MIBC:	
T2N0M0	23 (19)
T3 N0M0	19 (16)
T4/Any T N+/M+	2 (1.6)
Surgical procedure	
TUR	76(63)
Cystectomy	44(37)
Recurrence	
Number of patients with NMIBC	23(19)
Progression: Number of patients with NMIBC	8 (6.6)
Number of patients with MIBC	15 (12.5)
Survival Number of patients with MIBC	
Cancer-specific	
Alive	27 (22.5)
Deceased	17(14)
Overall survival	
Alive	25 (21)
Deceased	19 (16)

### Immunohistochemistry

Immunohistochemical staining was done on paraffin-embedded tissue, which had described in detail before[16]. Briefly, three-micrometer-thick sections were cut, using a rotation microtom. The sections were deparaffinized in xylene and rehydrated in graded alcohols and distilled water. After antigen retrieval with 0.01% EDTA (pH 8.0), endogenous peroxidase activity was blocked with 1% hydrogen peroxide in distilled water for 25 min followed by washing with distilled water and finally PBS + 0.1% Tween for 5 min. To bind nonspecific antigens, the sections were incubated with 1× Power Block (BioGenex) for 5 min. The primary antibodies for Snail, Slug, Twist, and E-cadherin were either polyclonal rabbit anti-Twist and anti-E-cadherin or polyclonal goat anti-Snail and anti-Slug, and purchased from Santa Cruz Biotechnology. Antibody dilution ranged from 1:50 to 1:150 in PBS for 30 min at 37°C. As negative control, sections were incubated with PBS instead of the primary antibody. This was followed by incubation with biotinylated antirabbit/antigoat immunoglobulin G (1:200; Santa Cruz Biotechnology) for 30 min at 37°C and peroxidase-conjugated avidin-biotin complexes (KPL) and 3,3'-diaminobenzidine (Sigma). The sections were then counterstained with Mayer's hematoxylin, upgraded alcohols, mounted, and analyzed by standard light microscopy.

### Evaluation of immunohistochemistry results

Immunohistochemical staining of Snail, Slug and Twist and E-cadherin was defined as detectable immunoreaction in perinuclear and/or cytoplasm. Expression of Snail, Slug and Twist was considered negative when no or less than 49% of the tumour cells were stained[16]. Cancer cells that were immunostained less than 10% staining were defined as having a reduced E-cadherin expression[17].

### Cell lines

The human bladder cancer cell lines (T24, HTB-3, HTB-1, HTB-2 and HTB-9) obtained from ATCC (Rockville, MD, USA). All the cell lines were maintained in RPMI 1640 medium supplemented with 10% FCS, 1% penicillin and streptomycin at 37°C in 5% CO_2_.

### Reverse transcription-polymerase chain reaction

Total RNA from the cell lines were obtained using RNeasy Mini kit(Qiagen, Tokyo, Japan) according to the manufacture's instructions and resuspended in 50 μL dimethylpyrocarbonate-treated water. RNA concentration was determined using a BioPhotometer (Eppendorf Scientific). Total RNA (2 μg) was primed with an oligo(dT) oligonucleotide and reverse transcribed with Moloney murine leukemia virus reverse transcriptase (Promega) and deoxynucleotide triphosphates (Sigma-Aldrich) according to the instructions of the manufacturer. First-strand cDNA was amplified with transcript-specific oligonucleotides using Ready-Mix Taq PCR Reaction Mix (Sigma-Aldrich). The primers (TIB Molbiol) for the respective genes were designed as follows: Slug (533 bp) 5'-GGTCAAGAAGCATTTCAAC-3'(sense) and 5'-GGTAATGTGTGGGTCCGA-3' (antisense);Snail (557 bp) 5'-CAACCCACTCAGATGTCAA-3' (sense) and 5'-CATAGTT AGTCACACCTCGT-3' (antisense); Twist (527 bp) 5'-GGGAGTCCGCAGTCTTAC-3' (sense)and5'-CCTGTCTCGCTTTCTCTTT-3' (antisense); E-cadherin (420 bp)5'-ATTC TGATTCTGCTGCTCTTG-3' (sense)and 5'-AGTAGTCATAG TCCTGGTCTT-3'(antisense);and β-actin (335 bp) 5'-TTCCTGGGCATGGAGTCCTGTGG-3' (sense) and 5'-CGCCTAGAAGCATTTGCGGTGG-3' (antisense). The condition of PCR for Slug were: initial denaturing at 95°C for 10 min, followed by 38 cycles of denaturing at 94°C for 60 s, annealing at 53°C for 60 s and extension at 72°C for 90 s. All PCR products were visualized by electrophoresis and ethidium bromide staining in 2% agarose gels. RT-PCR was performed in a triplicate.

### Western blotting analysis

For isolation of total protein, cells were washed twice with ice-cold PBS containing phosphatase inhibitor cocktail II (Sigma-Aldrich), scraped of the culture flask, pelleted by centrifugation, and lysed in buffer containing 10 mmol/L Tris (pH 6.8), 2 mmol/L EDTA (pH 8.0), 0.15 mol/L NaCL, 0.1% Brij 96, 0.1% NP40, 2 mmol/L phenylmethylsulfonyl fluoride, and 1× Protease inhibitor cocktail (Sigma-Aldrich). Protein was estimated using QuantiPro bicinchoninic acid assay kit (Sigma-Aldrich) according to the instructions of the manufacturer[16]. Ten micrograms of proteins were denatured at 95°C with sample buffer [0.125 mol/L Tris (pH 6.8), 4% SDS, 20% glycerol, 2% mercaptoethanol, 0.03 mmol/L bromphenol blue] for 5 min and separated by electrophoresis in 7.5% to 12% SDS-PAGE gels according to their molecular weight. Proteins were transferred onto a polyvinylidene difluoride membrane (Perkin-Elmer) and blocked for 2 h in blocking solution (5% nonfat dry milk in TBS containing 0.1% Tween 20) followed by 5% bovine serum albumin in TBS/Tween at room temperature on a rotating plate for 2 h. The membrane was then exposed to the primary antibody overnight at 4°C. The primary antibodies were the same we used for immunohistochemistry, and the dilution was 1:200 in Snail, Slug, Twist, and E-cadherin, and 1:500 in b-actin. After washing, the membranes were incubated for 1.5 h at room temperature with peroxidase-linked secondary antibody (Roche), and signals were detected using Lumilight Plus Western blotting kit reagents (Roche) according to the manufacturer's instructions and luminescence imaging (LAS-1000, Fujifilm).

### Statistical analysis

We used the χ^2 ^and Fisher's exact tests to evaluate the differences of staining of E-cadherin and Snail, Slug and Twist according to patient and cancer characteristics. The overall survival was defined as the time between the date of surgery and the last date of follow-up or date to death owing to bladder cancer. The progression-free survival was defined as the time interval between the date of surgery and the date of progression/recurrence or date of last follow-up. The curves were done using the Kaplan-Meier method with the log-rank test to assess the statistical significance. Cox proportional hazards analysis was used to determine the relative contribution of various factors to the risk of death, recurrence, and progression. *P *< 0.05 was considered as statistically significant. Analyses were performed with SPSS 10.00 software (SPSS, Chicago, IL).

## Results

### Expression of Snail, Slug, Twist and E-cadherin in human bladder cancer cell lines

**Figure 1:**
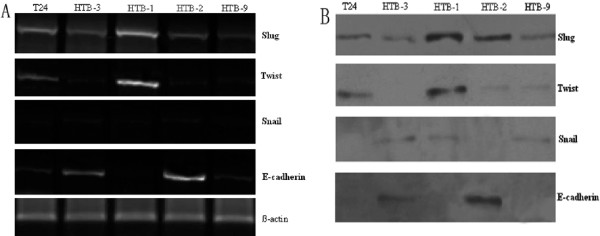
**Expression of Snail, Slug and Twist in five bladder cancer cell lines T24, HTB-1, HTB-2, HTB-3 and HTB-9**. The analysis of the relative mRNA and protein intensity of Slug, Snail and Twist compared with E-cadherin showed that bladder cancer cells with a high Slug and Twist expression had no or only low E-cadherin expression. In contrast, cells with low Slug and Twist expression had high expression levels of E-cadherin.

The expression of Snail, Slug, Twist and E-cadherin was analyzed at the mRNA and protein level by semiquantitative RT-PCR(Fig. 1A) and western blot (Fig. 1B) in the human bladder cancer cell lines T24, HTB-3, HTB-1, HTB-2 and HTB-9. Slug was expressed with different intensities in all five cancer cell lines. The undifferentiated HTB-1 and T24 cells had a strong mRNA and protein expression of Slug, whereas the other 3 cell lines showed only weak expression levels. Twist mRNA and protein was detected in HTB-1 and T24 cells, no appearant Twist mRNA and protein expression was found in other 3 cell lines. E-cadherin was detected in HTB-2, HTB-9 and HTB-3 cell lines. The most undifferentiated cell line HTB-1 and T24 cells showed no E-cadherin expression. Snail was not detectable in all five cancer cell lines. To verify intact RNA and protein, β-actin was used as a positive control.

#### Expression of Snail, Slug, and Twist in correlation with E-cadherin in human bladder cancer tissue

**Figure 2:**
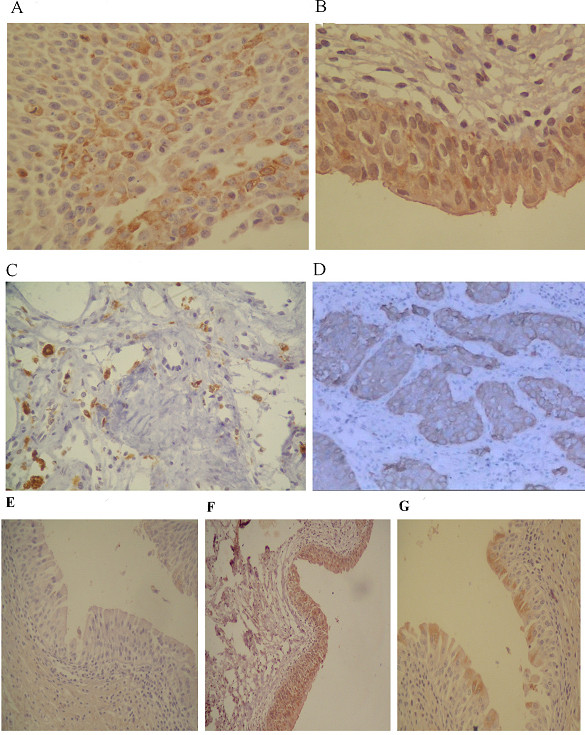
**Expression of Slug, Twist, Snail and E-cadherin in human bladder cancer and bankground tissue was determined by immunohistochemistry**. Staining of Snail(A), Slug(B), and Twist(C) was found in the cytoplasm as well as in the nucleus of tumor cells. Magnification, ×200. E-cadherin (*D*)expression was identified in the cell membrane and intensive in the cytoplasm. Magnification, ×200. No expression of Slug in bankground tissue(E), strong of Twist and Snail expression in bankground tissue (F-G).

Slug(A), Twist(B, F), Snail (Fig. 2C and 2G) in primary bladder cancer tissue were identified in the cytoplasm as well as in the nucleus of cancer cells. In general, staining for Slug and Twist was more intense than for Snail. E-cadherin expression in cancer cells was characterized by patterns with variable degrees of cytoplasmatic and membrane staining (Fig. 2D).

Immunohistochemistry showed that 44.2% (53/120) of human bladder carcinoma tissues and 38%(16/42) background tissue(G) expressed Twist(P = 0.156);62.5%(75/120) of human bladder Carcinoma tissues and 40%(17/42) background tissue(Fig. 2E) expressed Slug(P = 0.044); 15.8% (19/120) of human bladder carcinoma tissues and 76%(32/42) background tissue(Fig. 2F) expressed Snail(P = 0.016) and 25.8% (31/120) cases were low for E-cadherin expression in carcinoma tissues (Table 2). More patients with high Slug and Twist expression displayed low E-cadherin expression. Statistically significant correlations were found between Twist, Slug, and E-cadherin expression. No statistically significant correlations were found between Snail and E-cadherin expression(Table 3).

**Table 2:** Expression and Snail, Slug, Twist and E-cadherin in bladder cancer and background tissue

Variables	Positive expression(n)	Low expression(n)	**x**^**2**^	*P*
Slug			6.150	0.013
Cancer(120)	75	45		
Background(42)	17	25		
Snail			52.542	< 0.000
cancer(120)	19	101		
Background(42)	32	10		
Twist			0.469	0.493
cancer(120)	53	67		
Background(42)	16	26		

**Table 3:** Correlation between E-cadherin expression and Snail, Slug, and Twist expression in 120 cases of bladder cancer

	E-cadherin expression(n)		***X***^**2**^	*P*
**Slug **expression(n)	+(n = 89)	-(n = 31)		
+(n = 75)	64	11	13.016	0.000
-(n = 45)	25	20		
**Twist **expression(n)				
+(n = 53)	46	7	7.898	0.005
-(n = 67)	43	24		
**Snail **expression(n)				
+(n = 19)	11	8	3.523	0.061
-(n = 101)	79	22		

### Correlation between Snail, Slug, Twist and E-cadherin and clinicopathological parameters

There was a significant correlation between Twist overexpression and the tumor stage (*P *= 0.000)and grade(*P *= 0.000): superficial BT (Ta-1) (19 out of 76: 25%) and invasive BT (≥T2) (34 out of 44: 77.27%), LG (8 out of 41:19.51%), and HG (45 out of 79: 56.96%). The Twist immunoreactivity categorized into negative (< 2% of positive cells) vs. high expression was associated with several clinicopathological parameters: stage, grade, carcinoma in situ (CIS), progression(Table 3). In the pT1 BT group, the high-risk pT1b (lamina propria invasion)showed a Twist overexpression almost similar to invasive BT, explaining that the prognostic of both types of tumor is quite the same(date not showed). Of the 120 BT specimens, we demonstrated no correlation between Slug and Snail expression and stage and grade, but we demonstrated a correlation between E-cadherin expression and grade (p = 0.017): LMP (17 out of 18: 94.44%), LG (25 out of 41: 60.98%), and HG(64 out of 79: 81.01%) (Table 4). Only Twist was associated with clinicopathological parameters as progression(p = 0.035) (Table 4). It is interesting to note that Slug, Snail and E-cadherin had increased expression in node-positive tumors compared with node-negative tumors, these data reached significance(*P *= 0.012, P = 0.000, *P *= 0.040), respectively (Table 4). Twist was increased in node-positive tumors (node positive,10/18; node negative,43/102 p = 0.291), although the value was not significantly different.

**Table 4:** Relationship between the expression of Slug, Twist, Snail and E-cadherin and clinicopathological parameters in human bladder cancer

	Patients	Slug			Twist			Snail			E-cadherin		
	
Variables	*n*	+	-	*p*	+	-	*p*	+	-	*p*	+	-	*p*
Sex				0.493			0.557			0.664			0.824
Male	87	56	31		37	50		13	74		65	22	
Female	33	19	14		16	17		6	27		24	9	
Age (years)				0.257			0.523			0.947			0.845
≤ 70	64	43	21		30	34		10	54		47	17	
> 70	56	32	24		23	33		9	47		42	14	
Stage				0.171			0.000			0.986			0.874
pTa, pT1	76	51	25		19	57		12	64		56	20	
≥PT2	44	24	20		34	10		7	37		33	11	
Grade				0.082			0.000			0.789			0.017
LG	41	30	11		8	33		7	34		25	16	
HG	79	45	34		45	34		12	67		64	15	
Nodal involvement				0.012			0.291			0.000			0.040
yes	18	16	2		10	8		13	5		17	1	
no	102	59	43		43	59		6	96		72	30	
Recurrence(n = 76)				0.483			0.242			0.931			0.719
yes	23	15	8		12	11		5	18		16	7	
no	53	30	23		20	33		12	41		39	14	
Progression A(n = 76)				0.124			0.021			1.000			1.000
yes	8	3	5		6	2		3	5		7	1	
no	68	46	22		21	47		27	41		55	13	
Progression B(n = 44)													
yes	15												
no	29												
Death C(n = 44)				0.760			0.754			0.748			0.509
yes	17	9	8		11	6		5	12		13	4	
no	27	16	11		15	12		10	17		17	10	

### Correlation between proteins expression and BT recurrence

During the follow-up period (median follow-up time 30 months (1-89), the total number of cases in which recurrence was observed is 36(30%) between 2 to 62 months after initial diagnosis. We failed to demonstrate any significant association between this event and the clinicopathological data tested or the Slug, Twist, Snail and E-cadherin expression. Even in the univariate and multivariate analyses.

### Correlation of proteins expression with survival of patients with BT-Univariate analysis

Snail, Slug, Twist and E-cadherin is suggested to have a critical impact on progression and metastasis development as it positively influences the entrance of the tumor cells into the circulation (intravasation)[18-22]. To investigate the progression-free survival(PFS) or the overall survival (OS), we defined a time point of 36 months. During the time study, the 3-year PFS rates of patients who were positive (*n *= 53) and negative (*n *= 67) for Twist expression were 40% and 60%, positive (*n *= 75) and negative (*n *= 45) for Slug expression were 61% and 39%, and positive (*n *= 19) and negative (*n *= 101) for Snail expression were 15% and 85%, respectively (Table 4). In univariate analysis, positive expression of Twist, Snail and loss of E-cadherin expression, the stage, the grade, and CIS were significant predictors of short PFS. But positive expression of Slug was not significant predictors of short PFS(Table 5). For the 3-year OS rates, patients with Slug overexpression represented 34% and patients without,66%, patients with Twist overexpression represented 36% and patients without, 64%, and patients with Snail overexpression represented 18% and patients without, 82%(Table 5). Loss of E-cadherin expression, stage, grade, and CIS were also negative predictors of the OS (Table 5). We failed to demonstrate any significant correlation between OS and Twist, Slug and Snail,(Table 5).

**Table 5:** Univariate analyses of various clinicopathological parameters in relation to survival of patients with bladder tumor

Variables	Patients	Progression-free survival(PFS)		Overall survival(OS)	
	(*n *= 120)	5-year survival (%) (*n *= 103)	*P*-Value	5-year survival (%) (*n *= 61)	*P*-Value
Sex			0.051		0.363
Male	87	78(75.7%)		42(68.9%)	
Female	33	25(24.3%)		19(31.1%)	
Age (years)			0.108		0.591
≤ 70	64	58(56%)		34(55%)	
> 70	56	45(44%)		27(45%)	
Stage			0.175		0.016
pTa-T1	76	6871.8%		45(74%)	
≥PT2	44	3528.2%		16(26%)	
Grade			0.008		0.018
LG	41	40(38.8%)		27(38%)	
HG	79	63(61.2%)		34(62%)	
Slug			0.457		0.479
+	75	63(61%)		40(66%)	
-	45	40(39%)		21(34%)	
Twist			0.018		0.069
+	53	41(40%)		22(36%)	
-	67	62(60%)		39(64%)	
Snail			0.732		0.502
+	19	16(15%)		11(18%)	
-	101	87(85%)		50(82%)	
E-cadherin			0.000		0.005
+	89	86(83.5%)		52(85%)	
-	31	17(16.5%)		9(15%)	

### Multivariate analysis of prognostic variables in patients with BT

In this analysis, we only focused on markers of interest in this study. Doing a multivariate analysis with too many variables, even in 120 patients with BT, is bio-statistical nonsense. As stage, grade, or CIS are well-known prognostic factors in BT, we evaluated the expression of Snail, Slug, Twist and E-cadherin. In multivariate PFS analysis, Snail, Slug, Twist and E-cadherin were entered into the Cox proportional hazard analysis. Only Twist, Slug and E-cadherin expression retained significance as a prognostic factor of a short PFS (OR, 0.276; 95% CI, 0.090-0.841; *P *= 0.018, OR, 0.656, 95% CI, 0.215-2.003; *P *= 0.014, and OR, 23.208, 95% CI, 6.113-3.331; *P *= 0.000, respectively (Table 6). In multivariate OS analysis, only Slug and E-cadherin expression was an independently significant prognostic factor (OR, 0.409;95% CI, 0.017-0.140; *P *= 0.000; OR, 3.435;95% CI, 1.421-8.305, *P *= 0.005) (Table 6).

**Table 6:** Multivariate analyses of Snail, Slug, Twist and E-cadherin

Variables	Progression-free survival		Overall survival	
	OR (95% CI)	*P*-Value	OR (95% CI)	*P*-Value
Slug	0.656 (0.215-2.003)	0.457	0.409 (0.017-0.140)	0.000
Twist	0.276(0.090-0.841)	0.018	0.510(0.245-1.058)	0.069
Snail	0.858(0.221-3.777)	0.891	1.403(0.521-3.777)	0.502
E-cadherin	23.608(6.113-3.331)	0.000	3.435(1.421-8.305)	0.005

## Discussion

Recent studies have shown the role of Snail and Slug as strong repressors of E-cadherin gene expression in various cancer cell lines, including esophageal adenocarcinoma, lung, breast, endometrioid adenocarcinomas hepatoma HepG2 and human extrahepatic hilar cholangiocarcinoma, thus inducing tumor malignancy[23-28]. In addition, Twist is up-regulated in several types of epithelial cancers, including esophageal adenocarcinoma, malignant parathyroid neoplasia, hepatocellular carcinoma [29-31].

In our study, we have shown that the expression of Snail and Slug was significantly increased in human BT tissue than that of in background tissue. Moreover, the patients with strong E-cadherin expression showed no or less staining of Slug and Snail. A correlation between expression levels of Slug and E-cadherin was obvious in these human specimens(*P *= 0.013). which confirmed a previous study [32]. However, expression of Snail in BT showed no significant relation to the expression of E-cadherin. We have also shown that more patients with high Twist (46/53)expression displayed low E-cadherin expression (7/67), and high E-cadherin expression(43/67) displayed low Twist expression(24/53) in human BT tissue. There was an inverse relationship between Twist overexpression and loss of E-cadherin expression (*P *= 0.005), which confirmed a previous study [33,34]. We further studied the expression of Snail, Slug, Twist, E-cadherin in well established human BT cell lines. At the mRNA and protein level, BT cells with a high Slug and Twist expression had no or only weak E-cadherin expression, whereas no expression of Snail in BT cells was seen. Snail did not repress E-cadherin, neither at the RNA nor at the protein level. Comparing the expression levels of Twist, Slug and E-cadherin, there is evident that Slug and Twist is the strong repressor of E-cadherin. In undifferentiated BT cells (HTB-1 and T24), Slug and Twist completely repressed E-cadherin (Fig. 1). With increasing differentiation, Slug and E-cadherin or Twist and E-cadherin were coexpressed in BT cells (Fig. 1). This agrees with the fact that Slug and Twist is expressed at higher levels in poorly differentiated pancreatic cancer cell lines and that these tumors are more likely to grow invasive [35,36]. In contrast to Twist and Slug, Snail showed no expression in 84.2% of human BT tissues and in all five human BT cell lines. This was an interesting fact because several studies have shown an overexpression of Snail in a variety of different tumors [18,19,37]. However, the mechanism(s)involved therein have not been examined so far in BT.

Many studies have showed that Twist, Slug and Snail expression is related to the invasive proliferation of tumors and to be a poor prognostic factor in various human tumors. Our results showed that Twist expresse in human BT tissues was significantly correlated to the tumor stage, grade and progression (*P *= 0.000, *P *= 0.000 and *P *= 0.021, respectively);Snail was significantly correlated to nodal involvement *(P *= 0.000), but Snail was not involved in bladder tumor differentiation, stage, grade and progression;Slug was only significantly correlated to nodal involvement *(P *= 0.012). It would seem unnecessary to evaluate new progression marker in the very aggressive group of invasive BT of which the prognosis is pejorative, but the comparison of the molecular profile between superficial and invasive BT could allow individualizing a molecular marker of interest in order to treat earlier and more efficiently the high-risk of progression superficial bladder tumor. However, it has been demonstrated in human tumor cell lines that the forced expression of E-cadherin was not sufficient to reverse the process in Slug, Snail or Twist-expressing cells, and this result supports that Slug, Snail or Twist might modulate other important signaling pathways involved in tumor progression and metastasis development independently of E-cadherin expression[38-42]. Then, it strengthens the argument to justify both Slug or Snail or Twist expression and potential downstream targets as E-cadherin expression evaluation.

It has already been demonstrated that Slug, Snail or Twist overexpression was associated with poor outcome and shorter survival in patients with solid tumors [31-34]. Our findings support this hypothesis as E-cadherin and Twist expression were associated with poor survival both in univariate and multivariate analyses of all factors that influenced survival.

Franck Bruyere et al.[43]have found that high expression of Snail in superficial bladder tumors significantly predicts tumor recurrence in these patients. But we did not find any relationship between the expression of Snail and tumor recurrence. That we had a different way to evaluate the immunohistochemistry results may be the reasons.

The univariate analysis showed that high Snail expression, histologic grading, CIS and E-cadherin(*P *= 0.005)were statistically significant risk factors. The backward stepwise multivariate analysis showed that high Snail and Slug expression and E-cadherin were statistically significant independent risk factors. One mechanism of the link between Slug, Snail or Twist expression and poor prognosis may be the reducing of E-cadherin expression allowing cancer cells to migrate but it is probably not the only one[38-42].

Moreover, in our study, we noticed that some patients with high-risk superficial bladder tumors that High expressed Slug, Snail or Twist displayed distant metastasis within 2 years after initial diagnosis even if a cystectomy was performed. We also noticed that among patients who benefited from a cystectomy for invasive bladder tumor, 10/18 had lymph node metastasis with a bladder tumor high expressing Slug, Snail or Twist and without E-cadherin expression. These results suggest that although the prognostic value of Slug, Snail or Twist should be confirmed in a larger number of patients, its expression could be a useful marker for selecting patients with a high risk of a poor clinical outcome and for proposing a better therapy to them.

The inhibition of Slug, Snail or Twist action through interfering RNA (siRNA)or antisense transfer resulted in tumor metastasis or growth inhibition and increased sensitivity to the cytotoxic agents used in chemotherapy for solid cancers [29,44-46]These results strongly suggest the relation between EMT markers induction including Slug, Snail and Twist but also between anti-Slug, Snail or Twist treatment and improvement of bladder cancer chemotherapy.

In conclusion, the EMT regulatory proteins Slug and Twist are upregulated in human BT, whereas Snail is downregulated. Such disparate expression levels may contribute to the progression of tumors in BT, and this deserves further investigation. Our results highlighted the potential role of Twist, Snail and Slug as the prognostic factor in bladder cancer. They could be a very useful molecular marker of progression in BT. If our findings are validated by additional studies, Slug, Snail and Twist expression could be used as a predictive factor in bladder cancer but also as a novel target for clinical therapy. Identifying new molecular markers could also be the first step to accurately define a high risk-of-progression molecular profile in BT.

## Competing interests

The authors declare that they have no competing interests.

## Authors' contributions

QC and XS designed the experiments. KJ and XP carried out most of experiments and drafted the manuscript. ZM carried out the western blotting and KJ participated in statistical analysis and and interpretation of data. All authors read and approved the final manuscript.
